# Artificial intelligence in the early diagnosis of prostate cancer: from multimodal imaging to liquid biopsy

**DOI:** 10.3389/fonc.2026.1906810

**Published:** 2026-07-22

**Authors:** Jun Xie, Ziwei Yin, Ruisong Gao, Zerui Qiu, Qing Zhou

**Affiliations:** 1 The First Clinical School of Chinese Medicine, Hunan University of Chinese Medicine, Changsha, Hunan, China; 2 School of Integrated Chinese and Western Medicine, Hunan University of Chinese Medicine, Changsha, Hunan, China; 3 The First Hospital of Hunan University of Chinese Medicine, Changsha, Hunan, China

**Keywords:** artificial intelligence, early diagnosis, liquid biopsy, prostate cancer, radiomics

## Abstract

Prostate cancer (PCa) is the most prevalent malignant tumor in the urogenital system among men worldwide. Due to its subtle early symptoms and strong tumor heterogeneity, traditional diagnostic methods relying on a single prostate-specific antigen (PSA) initial screening and subjective imaging evaluations often lead to high false positives, overt biopsies, and missed small lesions. The rapid development of artificial intelligence (AI) provides innovative solutions to overcome these clinical bottlenecks. This article comprehensively reviews the application of AI in the early intelligent diagnosis of PCa. In the fields of ultrasound, magnetic resonance imaging (MRI), and positron emission tomography/computed tomography (PET/CT) imaging, AI significantly enhances the accuracy of target lesion identification. It achieves this by deep decoding high-dimensional quantitative features and effectively reducing subjective bias. In non-invasive liquid biopsy, AI-driven multi-omics networks have successfully addressed challenging screening blind spots, such as the PSA gray zone. In light of current challenges such as limited model generalization capability and the “black box effect” of algorithms, this article looks forward to the development prospects of constructing multimodal fusion models based on federated learning and explainable AI (XAI), aiming to promote the transition of PCa diagnosis and treatment from algorithm development to real clinical decision support.

## 1 Introduction

PCa originates from the epithelial cells of the male prostate and is one of the most common malignant tumors in the urinary system (1). According to global cancer statistics, its incidence ranks second among male malignancies and is the fifth leading cause of cancer-related death in men (2). The pathogenesis of this disease is highly complex, influenced by a combination of genetic susceptibility, endocrine metabolism, and environmental exposure (3). Due to the lack of specific clinical manifestations in early lesions, most patients are diagnosed only when they exhibit late symptoms such as urinary obstruction or pain from bone metastases, often missing the optimal time for intervention. Therefore, establishing an efficient and precise early screening and diagnostic system is crucial for reducing the burden of PCa and improving patient prognosis.

Currently, the clinical standard diagnostic pathway for PCa mainly consists of serum PSA initial screening, multiparametric MRI (mpMRI) assessment, and transrectal ultrasound (TRUS)-guided prostate biopsy (4). However, this traditional diagnostic paradigm reveals significant limitations in clinical practice. Firstly, although PSA has high diagnostic sensitivity, it has the characteristic of “organ specificity” rather than “tumor specificity”; benign prostatic hyperplasia or prostatitis also often leads to elevated levels, resulting in false positives, unnecessary invasive biopsies, and overdiagnosis (5). Secondly, the inherent blindness of traditional TRUS-guided systematic biopsies can easily result in missed diagnoses of small or deeply located lesions (6). Moreover, although the widespread use of mpMRI has significantly improved the accuracy of targeted biopsies, the interpretation of its images heavily depends on the subjective experience of radiologists, leading to considerable inter-reader variability (7). More critically, PCa exhibits profound heterogeneity in spatial distribution and molecular biology, making it difficult for traditional imaging methods to reliably distinguish between clinically insignificant (indolent) and clinically significant (aggressive) PCa (8). To overcome these inherent limitations and the high heterogeneity of the tumor microenvironment, there is an urgent clinical need for objective and non-invasive diagnostic tools. Although modern imaging genomics and molecular profiling can capture deeper pathological features, the high-dimensional datasets they generate far exceed human cognitive analytical capabilities. Therefore, the rapid advancement of AI technology provides an unprecedented opportunity to bridge this diagnostic gap.

**Figure 1:**
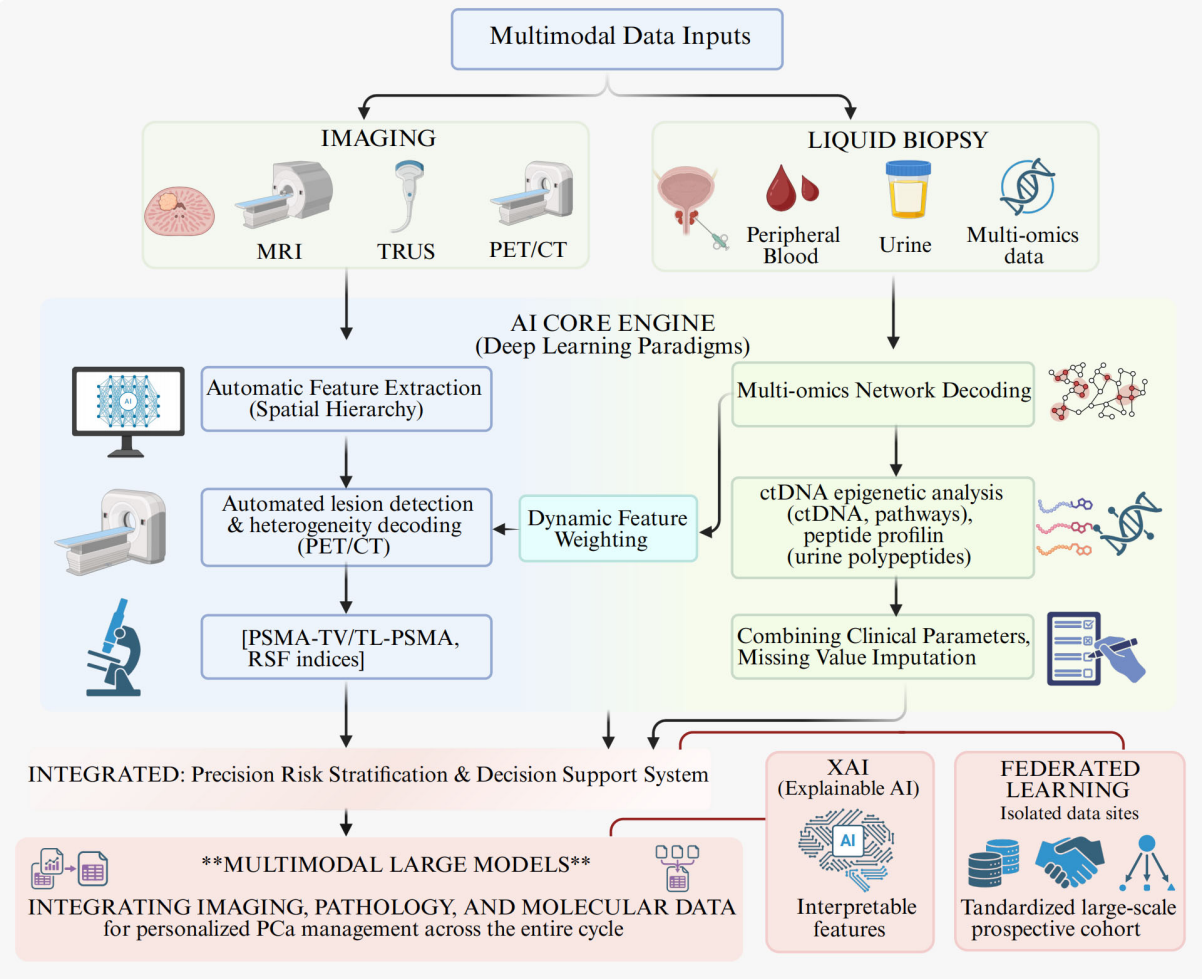
The overarching framework of AI-driven precision diagnosis in PCa. Multimodal inputs from imaging and liquid biopsies are processed by deep learning algorithms for automated lesion detection and complex multi-omics decoding. Ultimately, these datasets converge into “Multimodal Large Models.” Supported by XAI and federated learning, this integrated system aims to achieve precise risk stratification and personalized PCa management.

AI, as a cross-disciplinary field aimed at simulating human cognitive, reasoning, and multi-dimensional pattern recognition abilities, is reshaping the operational mechanisms of modern medical imaging at an unprecedented speed and depth (9). Within its vast technological landscape, machine learning (ML) and deep learning (DL) have emerged as the core engines for analyzing vast amounts of medical data. Traditional radiomics typically relies on classical ML algorithms such as random forests and support vector machines to model structured features such as morphological and texture features that are defined and extracted based on human expert experience. However, this approach of “manual feature engineering” is inevitably limited by the blind spots and biases of human cognition (10). As a higher-order evolution of ML, DL technology achieves “end-to-end” representation learning of unstructured medical images through the construction of multi-layer non-linear artificial neural network structures, thus enabling unprecedented precision in uncovering the microscopic pathological heterogeneity hidden beneath the surface of imaging pixels (11). In recent years, the underlying algorithmic architecture of medical imaging has undergone profound evolution. Convolutional neural networks (CNNs) and their derived models (such as U-Net) have fundamentally reduced the dependency on manual feature engineering by automatically extracting spatial hierarchical features, becoming the dominant paradigm for precisely delineating prostate lesions (12). Given the enormous potential of cutting-edge algorithms in decoding high-dimensional imaging features, the deep integration of AI and radiomics has become key to breaking through the traditional diagnostic and therapeutic bottlenecks in PCa. Thus, this paper comprehensively reviews the latest breakthroughs in AI network architectures within the field of PCa diagnosis. We critically analyze the numerous challenges hindering real-world clinical translation, such as the “black box effect” of algorithms and data silos, and we forecast future development trends, particularly the evolution towards XAI. By exploring the value of model interpretability in assisting clinical decision-making, we aim to provide a new technical perspective and solid support for precision treatment and personalized medicine in PCa (**Figure 1**).

## 2 Application of AI in imaging detection of PCa

Imaging examinations play an important role in the diagnosis, staging, and monitoring of PCa, providing essential information about the tumor’s location, size, morphology, and its relationship with surrounding tissues (13). Common imaging modalities include ultrasound, MRI, and PET/CT. The integration of AI and radiomics enables the extraction of sub-visual quantitative features, effectively compensating for the limitations of traditional methods. **Table 1** summarizes and compares representative AI architectures, performance, and clinical implications across these modalities.

**Table 1:** Summary and comparison of AI applications in different imaging modalities for PCa.

Image modalities	Researchers	Study design type	Sample size/Data details	AI architecture/Model	Validation method	Key performance metrics & results
Ultrasound	Li et al.	Retrospective single-center study	142 patients (1,696 2D prostate TRUS images)	FPN model	Random split-sample (Train/Val/Test) followed by randomized 5-fold cross-validation	Area under the curve (AUC): 0.934 Sensitivity: 0.829 Specificity: 0.966
	Sun et al.	Prospective 4-center diagnostic study	832 patients total	3D P-Net	Multi-cohort internal and independent external validation	AUC: 0.71-0.78 (vs. TRUS Likert) Biopsy rate: 40.3% → 34.0% Unnecessary biopsy: 38.1% → 25.8%
MRI	Mehralivand et al.	Retrospective, two-institution model development study	525 patients total Training: *n* = 368 (70%) Validation: *n* = 79 (15%) Test: *n* = 78 (15%)	AHNet vs. UNet	Split-sample validation	AHNet: Dice: 0.307, Sens: 74.4%, PPV: 47.8% UNet: Sens: 70.9%, PPV: 35.5%
	Grisales et al.	Retrospective, Algorithmic Architecture Development & Transfer Learning Feasibility Study	1,649 total scans: Prostate158 (*n* = 158, for pre-segmentation) and PI-CAI challenge (*n* = 1,491; 1,075 benign, 425 csPCa)	SAM-UNETR (SAM Transformer + UNETR Decoder)	Patient-locked split-sample validation (3:1:1 partition) benchmarking SAM-UNETR against three 2D architectures	Dice Score = 0.467 AUROC = 0.77 for csPCa segmentation
	Khosravi et al.	Retrospective multi-source model validation study	400 patients (228 in-house, 172 public DB)	AI-biopsy (with CAM)	Multi-source cross-validation.	Cancerous vs. Benign: AUC 0.89 High-risk vs. Low-risk: AUC 0.78
	Nißler et al.	Retrospective single-center MRMC non-inferiority study	105 consecutive men (Mean age: 66 ± 7 years) with suspected PCa.	AI-assisted bi-parametric MRI (AI-bpMRI)	Independent scoring by 3 radiologists with different levels of experience.	bpMRI non-inferior to mpMRI: GS≥3 + 4: ΔAUC = 0.03, 95%CI [-0.03,0.08], *p* = 0.37 GS≥3 + 3: ΔAUC = 0.04, 95%CI [-0.01,0.09], *p* = 0.142. Optimized bpMRI superior to conventional bpMRI (GS≥3 + 3): ΔAUC = 0.07, 95%CI [0.02,0.12], *p* = 0.004
	Saha et al.	International paired confirmatory study	10,207 MRI examinations from 9,129 patients total	State-of-the-art Grand Challenge AI system	Two-arm external validation against 62 radiologists (*n* = 400) and multidisciplinary routine practice (*n* = 1,000)	AI AUROC = 0.91 vs. Radiologist AUROC = 0.86 False positives reduced by 50.4% Overdiagnosis reduced by 20.0%
PET/CT	Leung et al.	Retrospective classification model development study	267 total men with PCa, contributing 3,794 total lesions.	DL + radiomics	Lesion-level split-sample validation	Lesion-level AUC: 0.92 Patient-level AUC: 0.85
	Huang et al.	Retrospective, two-center model development study	116 total patients with metastatic PCa experiencing post-prostatectomy recurrence	3D U-Net Framework	5-fold cross-validation (*n* = 78) followed by internal (*n* = 19) and external (*n* = 19) testing	Internal/external f1-score: 0.824/0.837 tumor burden metrics: R^2^≥0.991
	Janbain et al.	Multinational retrospective	1,029 post-operative patients with sRT	RSF model	Multinational internal & external validation	C-index: 0.54-0.91

### 2.1 Ultrasound examination

Ultrasound examination, particularly transrectal ultrasound (TRUS), serves as the bedrock for the initial screening and real-time biopsy guidance of PCa by visualizing glandular boundaries and lesion echogenicity (14). However, traditional TRUS suffers from relatively low soft-tissue contrast and resolution, often hindering the precise delineation of tumor staging and sub-centimeter aggressive foci. To circumvent these acoustic limitations, computational strategies have transitioned from traditional pixel-intensity analysis to multi-scale contextual feature modeling. Representing a pivotal advancement in structural spatial encoding, Li et al. (15) developed a DL model leveraging a feature pyramid network (FPN) based on static TRUS imaging to predict PCa. By extracting and fusing low-level high-resolution textures with high-level semantic abstractions, the FPN model achieved an outstanding AUC of 0.934 (sensitivity: 0.829, specificity: 0.966), significantly outperforming both standard clinical baselines (*p* < 0.05) and experienced radiologists (AUC: 0.667). Crucially, the mathematical superiority of this FPN framework over standard single-scale CNNs stems from its intrinsic multi-level lateral connections, which effectively preserve localized micro-echo variations typical of diffuse malignancies. Nevertheless, a major cross-study methodological divergence persists: while Li et al. reported exceptionally high diagnostic metrics, their model relied on curated single-center static frames, a constraint that heavily abstracts away the real-time, dynamic nature of clinical sonography and increases vulnerability to cross-vendor machine noise.

To resolve this static limitation and capture the full spatial continuity of the gland, recent architectural innovations have scaled from 2D static feature representations to 3D/temporal cinematic analysis. Addressing this gap, Sun et al. (16) developed a high-performance three-dimensional convolutional neural network (3D P-Net) engineered to identify clinically significant PCa (csPCa) directly from dynamic TRUS videos. Rather than scanning isolated planes, the 3D P-Net processes continuous spatio-temporal video tensors, allowing the network to filter out transient out-of-plane acoustic artifacts that frequently mimic malignant hypo-echogenicity on static 2D scans. Consequently, the 3D P-Net substantially outperformed the traditional TRUS 5-point Likert scoring system (AUC: 0.71–0.78) and achieved parity with the gold-standard mp-MRI PI-RADS v2.1 scoring system interpreted by senior radiologists (AUC: 0.83–0.86). More importantly, this transition from static spatial extraction to temporal consensus directly translated into a tangible clinical downgrade in overdiagnosis: the overall biopsy rate decreased from 40.3% to 34.0%, and the unnecessary biopsy rate dropped precipitously from 38.1% to 25.8%. When benchmarking these two paradigms, a critical academic trade-off emerges between Li et al.’s FPN approach and Sun et al.’s 3D P-Net framework. The 2D FPN architecture demonstrates an acute sensitivity for localized, high-contrast lesions within static sectors, yet as a static frame-based approach, it remains highly susceptible to false positives induced by user-dependent probe-pressure variations and out-of-plane acoustic artifacts. Conversely, the 3D video-based network sacrifices a degree of fine-grained, single-pixel boundary sharpness but successfully leverages inter-frame temporal redundancy to smooth out motion artifacts, rendering it far more robust for reducing unnecessary invasive procedures. In summary, AI-driven ultrasound models demonstrate profound clinical translational value by elevating a low-cost, frontline modality to diagnostic parity with expensive, contrast-dependent mp-MRI frameworks. However, the high operator dependence and acoustic shadowing inherent to TRUS still restrict the universal generalization of these models. In the future, the deep integration of standardized automated image acquisition with cross-platform multicenter validation will be mandatory to entirely eliminate subjective scanning biases, unlocking the full potential of AI-ultrasound as an accessible, high-throughput frontline guardian against PCa overdiagnosis.

### 2.2 MRI diagnosis

mpMRI is currently the “gold standard” for non-invasive diagnosis of PCa, but overcoming the subjectivity of interpretation remains a pressing clinical challenge (17, 18). AI architectures address this bottleneck by automating high-dimensional feature extraction. Early DL explorations primarily focused on raw pixel-level analysis to segment or detect lesions. For instance, Mehralivand et al. (19) developed an automated PCa detection system trained on data from two institutions, demonstrating that an AHNet model achieved a Dice coefficient of 0.307, a sensitivity of 74.4%, and a positive predictive value (PPV) of 47.8%, outperforming the standard UNet (sensitivity: 70.9%, PPV: 35.5%). Although these metrics validate the capability of convolutional networks to enhance sensitivity, the critically low PPV and baseline Dice scores underscore an inherent bottleneck: raw pixel-level analysis often struggles with dense spatial context, leading to a high false-positive rate. This limitation highlights an urgent clinical need to integrate secondary filtering mechanisms and more robust semantic feature representations into model architectures.

To enhance boundary definitions at the sub-visual level, recent methodologies have integrated global contextual awareness via foundation model backbones. Grisales et al. (20) pioneered this transition with SAM-UNETR, fusing the Vision Transformer (ViT) encoder from the Segment Anything Model (SAM) with a UNETR-derived convolutional decoder. Incorporating anatomically guided zonal preprocessing, SAM-UNETR advanced the csPCa lesion-level DSC to 0.467. Structurally, this incremental improvement (DSC 0.467 vs. Mehralivand’s 0.307) underscores the benefit of the Transformer’s global attention mechanism in capturing long-range dependencies across multi-parametric sequences. Nevertheless, a critical methodological challenge persists: despite large-scale natural image pre-training, foundation models like SAM still deliver low absolute overlap metrics (DSC < 0.50) when tasked with ill-defined, infiltrative tumor lesions rather than distinct structural organs. This performance ceiling stems from the fact that SAM lacks intrinsic domain-specific visual priors for diffuse, contrast-attenuated malignancies, highlighting the ongoing clinical necessity for deep semantic filtering frameworks (such as advanced self-configuring architectures like nnU-Net) to master fine-grained lesion delineation.

Beyond pure structural segmentation, another critical dimension in translating AI to radiological workflows is mitigating the “black box” nature of deep neural networks to build clinician trust. To alternatively resolve the false-positive dilemma while enhancing interpretability, frameworks featuring class activation mapping (CAM) have emerged. Khosravi et al. (21) developed the “AI-biopsy” system, which achieved an AUC of 0.89 in cancerous/benign differentiation and 0.78 in risk stratification. Unlike pure segmentation models, AI-biopsy integrates a fully automated web interface with real-time visual explanations, mapping out the precise feature matrices driving its risk scores. This focus on clinical utility and decision reproducibility presents an alternative avenue for biopsy reduction. Crucially, as algorithmic representations grow more sophisticated, they unlock possibilities not only for software integration but also for simplifying imaging protocols themselves. Addressing the scanning complexity and contrast-agent risks of full mpMRI, Nißler et al. (22) conducted a retrospective evaluation of an AI-assisted biparametric MRI (AI-bpMRI) framework. Their results proved that AI-bpMRI significantly outperformed conventional bpMRI and achieved non-inferiority to the gold-standard contrast-enhanced mpMRI in detecting index lesions, specifically clinically significant cases defined by Gleason scores ≥ 3 + 4 (ISUP Grade Group ≥ 2). This suggests that high-order feature extraction can effectively compensate for the lack of dynamic contrast-enhanced sequences, providing a cost-effective, contrast-free alternative tailored for primary community screenings.

Ultimately, validating whether these modular innovations—ranging from segmentation transformers to contrast-free screening—can truly withstand real-world clinical heterogeneity requires moving beyond single-center cohorts toward large-scale, prospective-grade evidence. Bridging this gap, Saha et al. (23) conducted the PI-CAI international multicenter confirmatory study, benchmarking a state-of-the-art AI system against global radiological expertise using over 10,000 MRI examinations tied to a rigorous 5-year follow-up reference standard. In a controlled multireader setting, the AI system exhibited clear superiority, achieving an AUROC of 0.91 against the average AUROC of 0.86 pooled from 62 international radiologists, while cutting false positives by 50.4% and overdiagnosis by 20.0% at identical sensitivities. However, a profound nuance emerged when evaluated against routine, real-world multidisciplinary practice: the AI’s superiority transitioned into non-inferiority, yielding a comparable performance to the clinical standard of care where human readers utilized patient history and peer consultation. This performance shift reveals a vital clinical reality: while standalone AI easily outpaces the isolated human eye by eliminating subjective bias, it does not yet fully surpass the collective intelligence of a fully optimized human multidisciplinary team. Consequently, the current consensus dictates that rather than acting as a total replacement, these advanced systems are best positioned as frontline triage and workload-reduction tools, whose long-term clinical applicability must be further validated through prospective clinical implementation.

### 2.3 PET/CT diagnosis

The widespread application of prostate-specific membrane antigen (PSMA) PET/CT has advanced the imaging evaluation of PCa to a molecular targeted level. However, the immense volume of three-dimensional metabolic data coupled with the profound physiological tracer uptake in non-target organs (such as salivary glands, kidneys, and bowel) often pose significant challenges for clinical visual interpretation (24). The intervention of AI is uniquely suited to this complex scenario: DL models can efficiently decode vast high-dimensional metabolic heterogeneity features, accurately decoupling pathological signals from background noise caused by physiological excretion. This not only reduces the cognitive burden on physicians but also significantly enhances the diagnostic precision for identifying small, low-expression target lesions (25).

To address this lesion-to-background discrimination challenge, algorithmic strategies have bifurcated into hybrid engineered feature frameworks and end-to-end deep convolutional networks. Emphasizing the former paradigm, the Leung team’s retrospective study developed an AI framework that structurally integrated DL representations with hand-crafted radiomic features for automated analysis of PSMA PET images. By leveraging this multi-level feature fusion, the framework successfully distinguished malignant PCa lesions from complex physiological confounding uptakes, achieving an AUC of 0.92 at the lesion level and 0.85 at the patient level, while establishing a high degree of concordance with the standardized PSMA-RADS reporting system (26). Conversely, relying purely on hierarchical spatial convolutions without manual feature engineering, Huang et al. (27) deployed an end-to-end 3D U-Net model engineered for [^68^Ga]Ga-PSMA-11 PET/CT to automatically segment systemic metastases in post-prostatectomy recurrent patients. This spatial-dense architecture demonstrated exceptional lesion recognition, yielding F1-scores of 0.824 and 0.837 across internal and external validation sets, respectively. Crucially, the automatically quantified systemic tumor burden metrics—PSMA tumor volume (PSMA-TV) and total lesion PSMA (TL-PSMA)—exhibited near-perfect linearity with expert consensus annotations (R^2^≥0.991). From a methodological perspective, the robust F1-scores achieved by Huang et al. represent a significant architectural milestone over traditional 2D models; however, a subtle yet critical consensus divergence persists in current literature. While 3D U-Net variations excel at volumetric segmentation of bulky nodal or bone metastases, their localized spatial pooling layers often suffer from feature dilution when detecting sub-centimeter or low-avid metastatic lesions, a limitation that necessitates the ongoing exploration of hybrid architectures like those utilized by Leung’s team to maintain multi-scale diagnostic sensitivity.

After achieving robust image segmentation and volumetric tumor burden quantification, the clinical utility of AI combined with PSMA PET/CT naturally extends from cross-sectional diagnostics to longitudinal, personalized treatment guidance and long-term prognostic forecasting. Transitioning from spatial analysis to temporal survival analytics, a landmark multinational retrospective study involving 1,029 patients evaluated the efficacy of PSMA-PET-guided salvage radiotherapy (sRT) in post-operative biochemically recurrent PCa patients. This investigation led to the development of a random survival forest (RSF) machine learning prognostic model. Following rigorous internal and external validation, the RSF model demonstrated superior predictive performance, yielding a C-index ranging from 0.54 to 0.91, which significantly outperformed traditional clinical nomograms (28). The intrinsic mathematical superiority of the RSF architecture over conventional Cox proportional hazards models stems from its capacity to inherently capture high-order, non-linear interactions and complex multi-variable correlations among PSMA avidities, anatomical locations, and clinical staging without rigid baseline assumptions. Nevertheless, the wide fluctuation in the reported C-index (0.54 to 0.91) highlights a critical vulnerability in current radiomics-based survival modeling: the model’s prognostic accuracy is heavily dependent on the clinical heterogeneity of the external validation cohorts and variations in scan acquisition protocols across institutions. Overall, the deep integration of AI with PSMA PET/CT has successfully facilitated the transition from subjective, qualitative lesion analysis to objective, high-dimensional quantitative profiling, effectively closing the clinical loop from initial lesion screening to long-term therapeutic response prediction for patients with recurrent PCa.

## 3 Application of AI in liquid biopsy and multi-omics profiling for PCa

Ideal liquid biomarkers, such as molecular signals derived from peripheral blood or urine, are regarded as the optimal carriers for non-invasive early screening of PCa due to their non-invasiveness and ease of accessibility. However, despite the emergence of novel indicators like the Prostate Health Index and urine PCA3, there is currently no single biomarker with sufficient sensitivity and specificity to independently replace tissue biopsy (29, 30). The currently most widely used single PSA screening is limited by a very high false positive rate, inevitably leading to overdiagnosis and unnecessary biopsy-related complications (31). To overcome the translational bottleneck faced by single biomarkers, AI-driven combined diagnostic models provide a disruptive solution. At the foundational clinical modeling level, ML algorithms can dynamically assign weights and perform nonlinear modeling on multidimensional conventional parameters such as digital rectal examination (DRE) and PSA-derived indicators, reshaping early risk stratification without incurring additional costs.

A more prominent breakthrough lies in the deep integration of AI with high-throughput omics technologies, marking a profound leap in liquid biopsy from “single molecular quantification” to “decoding complex multi-omics networks.” Distinct biological layers exhibit varied clinical execution potentials and distinct algorithmic affinities. Urinary proteomics, for instance, offers direct insights into real-time phenotypic changes. Frantzi et al. (32) utilized capillary electrophoresis-mass spectrometry on natural urine samples from 970 patients, employing a Support Vector Machine (SVM) algorithm to develop an 181-endogenous-peptide profile (AUC = 0.81) that eliminates the need for prior DRE. The choice of SVM here is technically well-suited for handling high-dimensional, sparse proteomic features by identifying optimal separating hyperplanes, though it remains highly sensitive to platform-dependent measurement noise. Conversely, epigenetic alterations often precede gross transcriptional shifts, presenting higher stability for early capture. Lleshi et al. (33) trained an ML model on enriched methylated cell-free DNA fragments, demonstrating robust performance in detecting metastatic and localized PCa (AUCs of 0.96 and 0.74, respectively). This noticeable performance gap between localized and metastatic detection highlights a common divergence in current literature: epigenetic signals in early-stage localized tumors are frequently diluted in peripheral blood, demanding more sensitive, deep-learning-based denoising architectures to capture faint early-stage signatures. For highly dynamic functional readouts, transcriptomics offers massive utility. Kim et al. (34) developed the “PCASSO” framework, applying a Gradient Boosting algorithm to 20 urinary RNA biomarkers from 163 samples to construct an optimized 9-biomarker model boasting an exceptional AUC of 0.99. This ensemble tree-based architecture inherently excels here because it effectively captures complex feature interactions and non-linear boundaries among a tight selection of correlated transcriptomic markers, maintaining high accuracy even within the clinically ambiguous PSA gray zone (3–10 ng/mL). While single-omic models show promise, they often fail to capture the holistic biology of PCa, leading to diagnostic blind spots. Joint multi-analyte profiling addresses this by exploiting biological cross-talk across heterogeneous data layers. Miller et al. (35) pioneered a comprehensive approach concurrently isolating extracellular vesicle (EV) RNA and cfDNA from the urine of 106 patients. By leveraging high-depth sequencing alongside machine learning to profile gene expression, splice variants, and over 18,000 differentially methylated bases, their joint epigenetic-transcriptomic model achieved an outstanding AUC of 0.92—significantly outperforming both PSA alone (AUC = 0.52) and mpMRI (AUC = 0.65). This directly demonstrates that multi-omic feature synergy can resolve the clinical gray-zone dilemmas that confound single-modality imaging.

The ultimate paradigm shift involves fusing these high-dimensional molecular networks with spatial imaging features. Xu et al. (36) addressed the clinical dilemma of excessive biopsies within the 4–10 ng/mL PSA gray zone by developing a multimodal DL model that integrates multi-parametric MRI (mpMRI) sequences with clinical parameters, achieving an AUC of 0.913 and a specificity of 90.9%, which could potentially reduce unnecessary biopsies by 40–50%. However, scaling this integration to connect high-dimensional multi-omics with radiomic features introduces severe computational challenges: first, the “curse of dimensionality” and overfitting, where combining tens of thousands of genomic/epigenetic features with thousands of radiomic textures creates an asymmetric data matrix where features vastly outnumber the sample size (*p* ≥ *n*); second, cross-modality heterogeneity, where discrete or continuous molecular counts and continuous spatial pixel intensities possess fundamentally incompatible statistical distributions, rendering early-fusion (simple concatenation) ineffective; third, real-world data incompleteness, where patients rarely possess a complete matrix of imaging and multi-omic data simultaneously. To resolve these computational hurdles, advanced architectures are transitioning toward intermediate-fusion or late-fusion strategies. Against the backdrop of massive real-world cohorts where incomplete clinical parameters are a baseline reality, incorporating missing value imputation algorithms into deep neural networks has proven vital (37).

Effective AI-driven multi-omics integration for prostate cancer risk stratification requires harmonization across heterogeneous data layers, including somatic mutation profiles, cell-free DNA methylation patterns, RNA expression signatures, and proteomic biomarkers. Graph-based neural networks and attention-based transformer architectures have shown particular promise in modeling the complex inter-omic dependencies that underlie aggressive disease phenotypes. When combined with radiomic features extracted from mpMRI, such multimodal molecular-imaging frameworks may offer a more complete biological portrait of tumor biology than either approach alone (38). Prospective validation in diverse, multicenter cohorts is essential before these tools can enter routine clinical decision-making. In summary, the deep integration of AI with multidimensional liquid biopsy and imaging is fundamentally reshaping the landscape of early, non-invasive PCa diagnosis. By efficiently decoding data from routine clinical parameters up to high-dimensional omics networks, intelligent warning models have successfully addressed the screening blind spots of single biomarkers. However, while algorithm-driven methodological innovation demonstrates vast potential, the field must systematically reduce the financial cost and technical complexity of multi-omic sequencing in the future to truly advance the comprehensive implementation of precise PCa screening.

## 4 Summary and outlook

In summary, AI has demonstrated transformative advantages in both multimodality imaging recognition (ultrasound, MRI, and PSMA PET/CT) and high-throughput multi-omics decoding for PCa liquid biopsies. By autonomously extracting sub-visual, high-dimensional quantitative features, AI frameworks effectively mitigate the inherent subjective biases of traditional radiological interpretation while facilitating dynamic, personalized risk stratification within critically ambiguous clinical scenarios such as the PSA gray zone.

However, transitioning these algorithmic innovations from controlled computational benchmarks to real-world clinical deployment remains hindered by rigorous cross-disciplinary barriers. At the data level, the current literature is heavily dominated by single-center, retrospective cohort designs. The pervasive technical heterogeneity in imaging scanner architectures, acquisition protocols, and multi-omics sequencing platforms across institutions frequently precipitates catastrophic model overfitting, thereby undermining algorithmic generalization across diverse patient populations. More importantly, this translation bottleneck is further compounded by the stringent regulatory landscape governing Software as a Medical Device (SaMD). In the United States, AI-driven diagnostic tools face bifurcated regulatory clearance pathways under the Food and Drug Administration, navigating either the 510(k) premarket notification predicated on establishing substantial equivalence to legally marketed predicate devices, or the more demanding *De Novo* classification and Premarket Approval pathways requiring exhaustive prospective safety profiles for novel, high-risk clinical tasks. Concurrently, in the European Union, the stringent Medical Device Regulation (MDR 2017/745) (39) has significantly elevated the criteria for CE marking, mandating rigorous, continuous clinical evaluation portfolios and robust post-market surveillance that create formidable hurdles for rapid software commercialization. This regulatory friction is exacerbated by a fundamental disconnect in validation methodologies: while developers traditionally rely on static diagnostic metrics such as AUROC and F1-scores, these mathematical indices fail to measure actual clinical utility or the downstream consequences of test-directed interventions. To bridge this evaluation gap, recent validation paradigms have emphasized the integration of Decision Curve Analysis (DCA). By incorporating patient risk thresholds and mathematical net benefit functions, DCA effectively quantifies the clinical value of AI-guided strategies, capturing whether an algorithm truly minimizes unnecessary, invasive prostate biopsies or costly overdiagnosis relative to standard-of-care pathways.

In the future, navigating these regulatory and clinical hurdles will define the core trajectory for the intelligent management of PCa. Methodologically, the deployment of privacy-preserving paradigms such as federated learning and secure multi-party computation will unlock distributed institutional data silos without compromising patient privacy, facilitating the creation of massive, global prospective validation cohorts. Furthermore, the maturation of XAI frameworks will demystify the neural “black box” through feature attribution and semantic visualization, aligning algorithmic causal logic with underlying histopathological and pathophysiological mechanisms to cultivate institutional clinician trust. Ultimately, fusing cross-modal imaging, digital pathology, and longitudinal multi-omics profiles into cohesive “multimodal medical foundation models” will catalyze the transition of AI from isolated auxiliary software into an interconnected, regulatory-compliant, and clinically validated decision-support ecosystem, laying a resilient intelligent foundation for the precision management of PCa across the entire patient care continuum.
